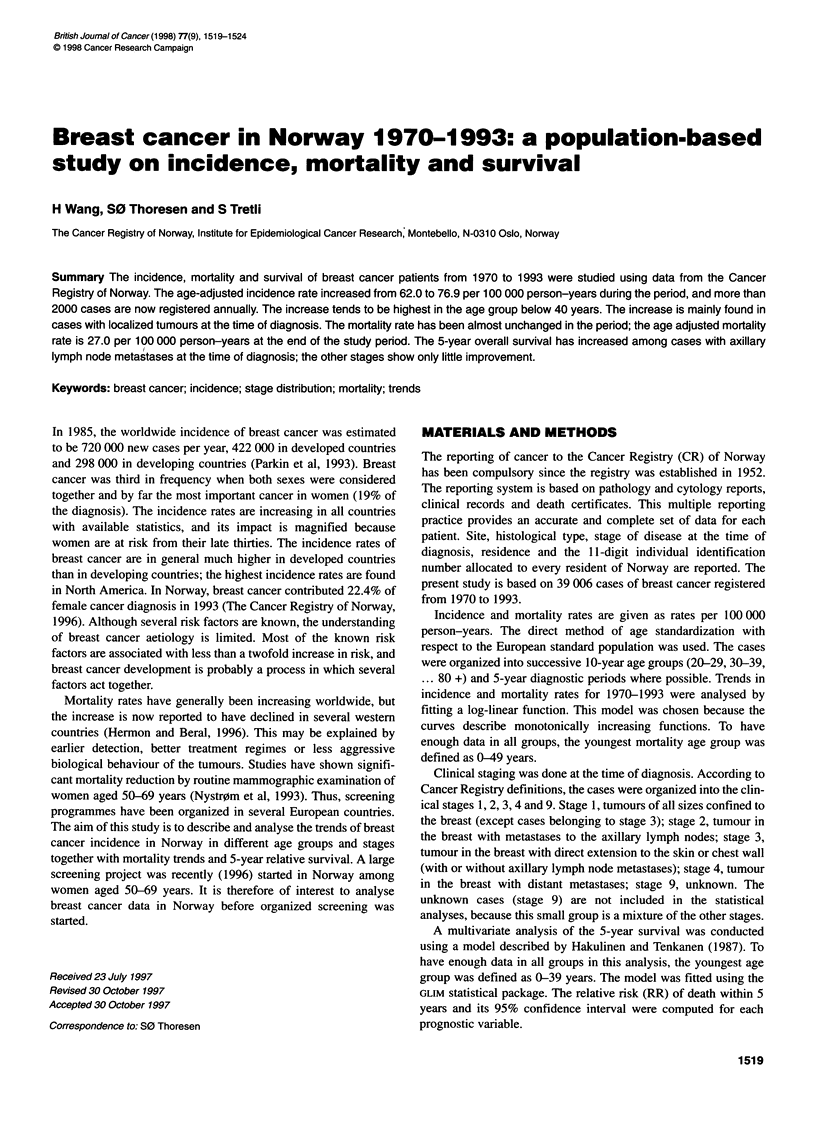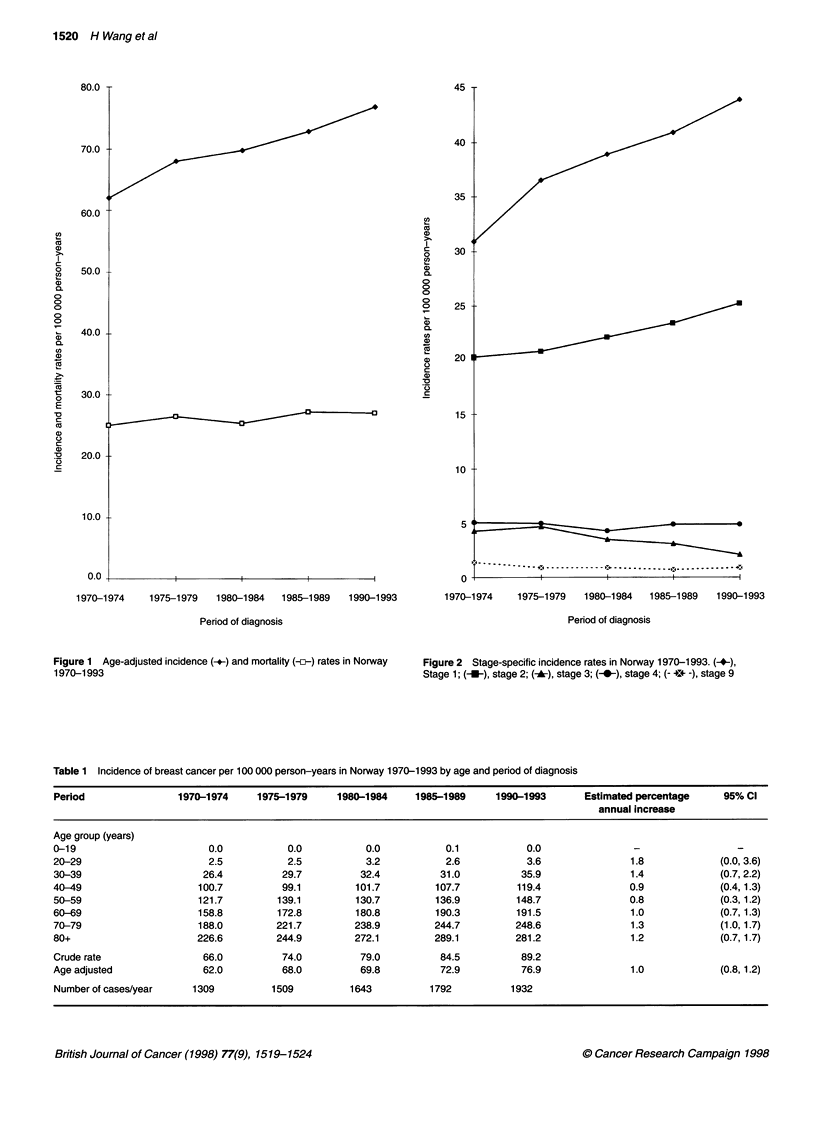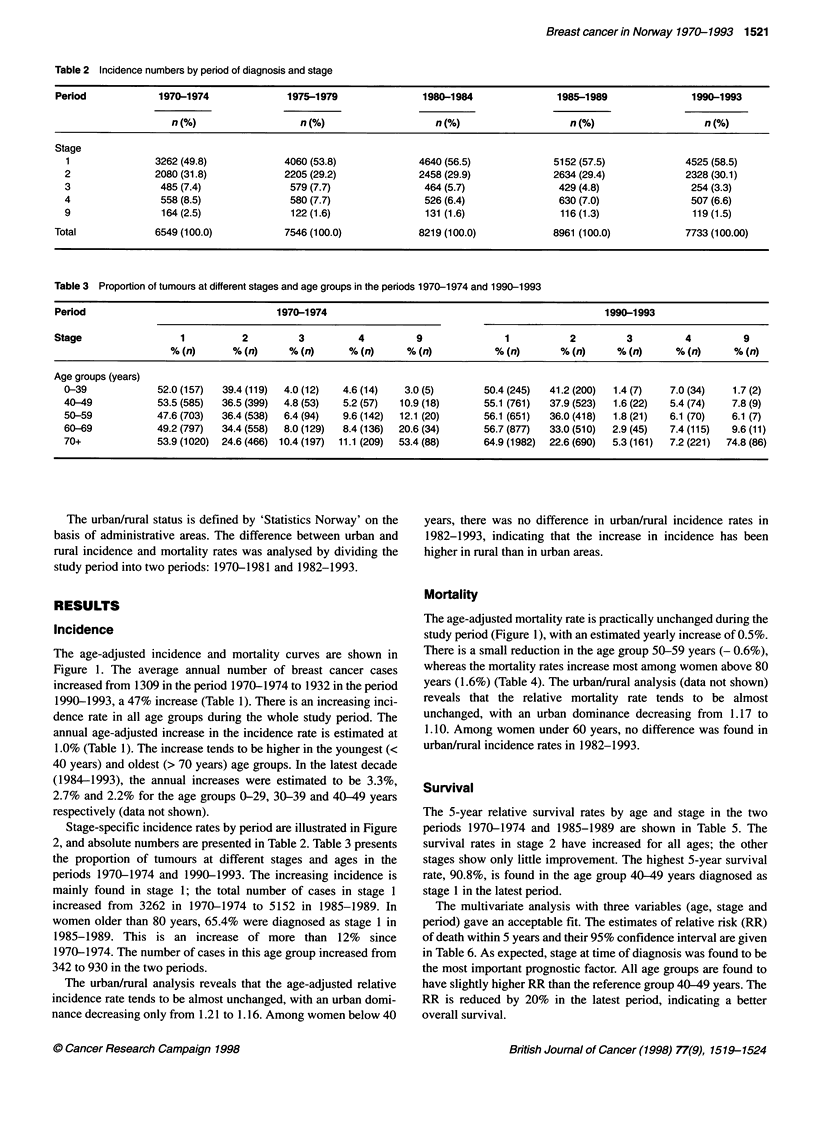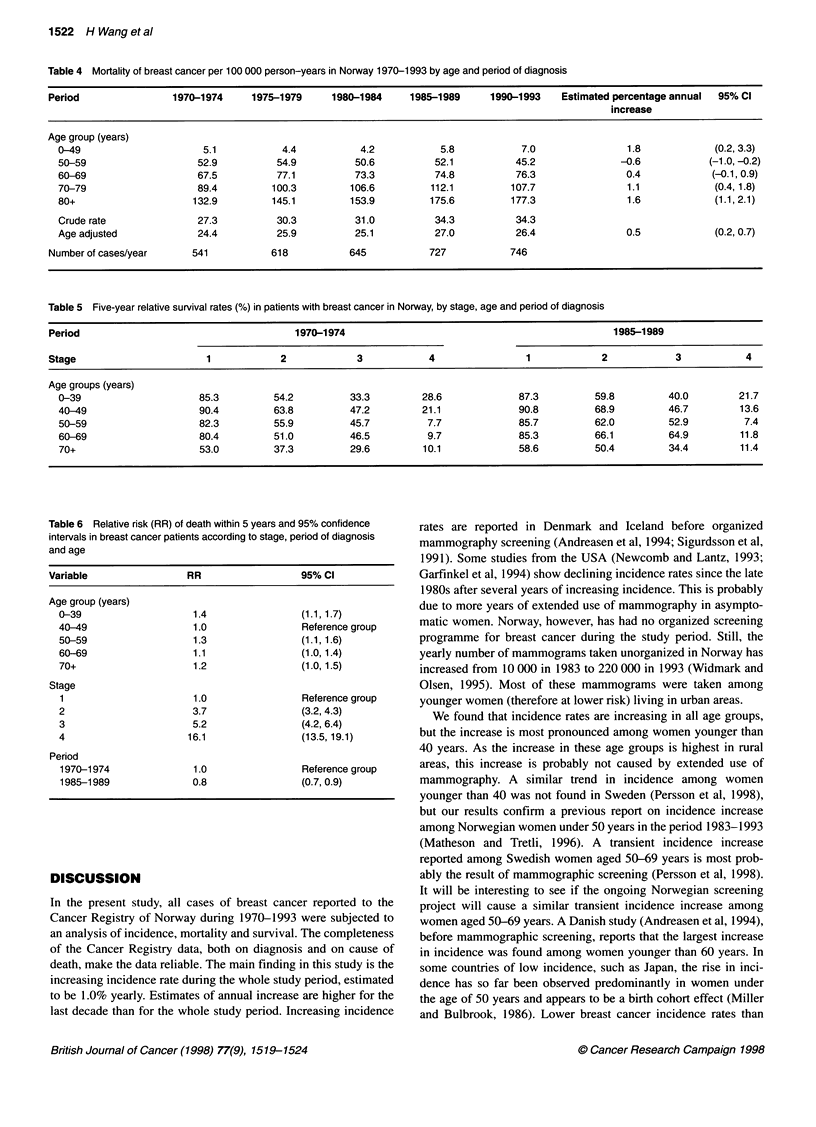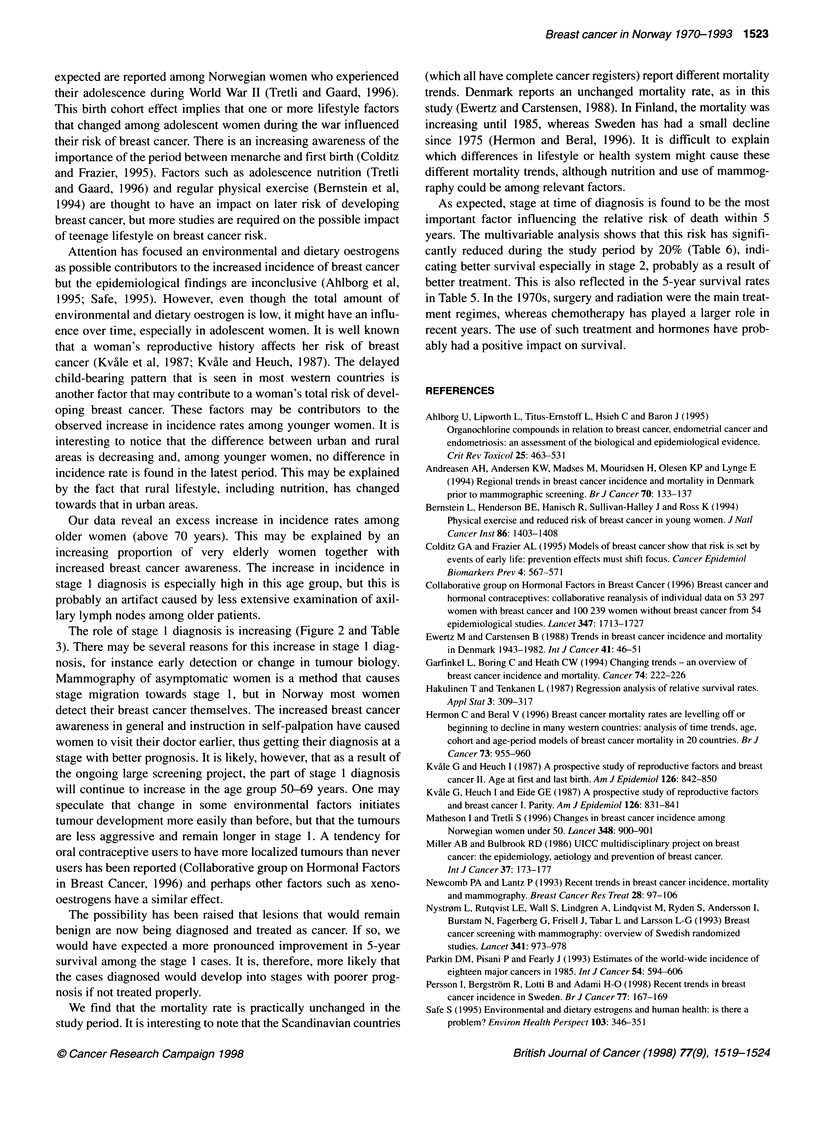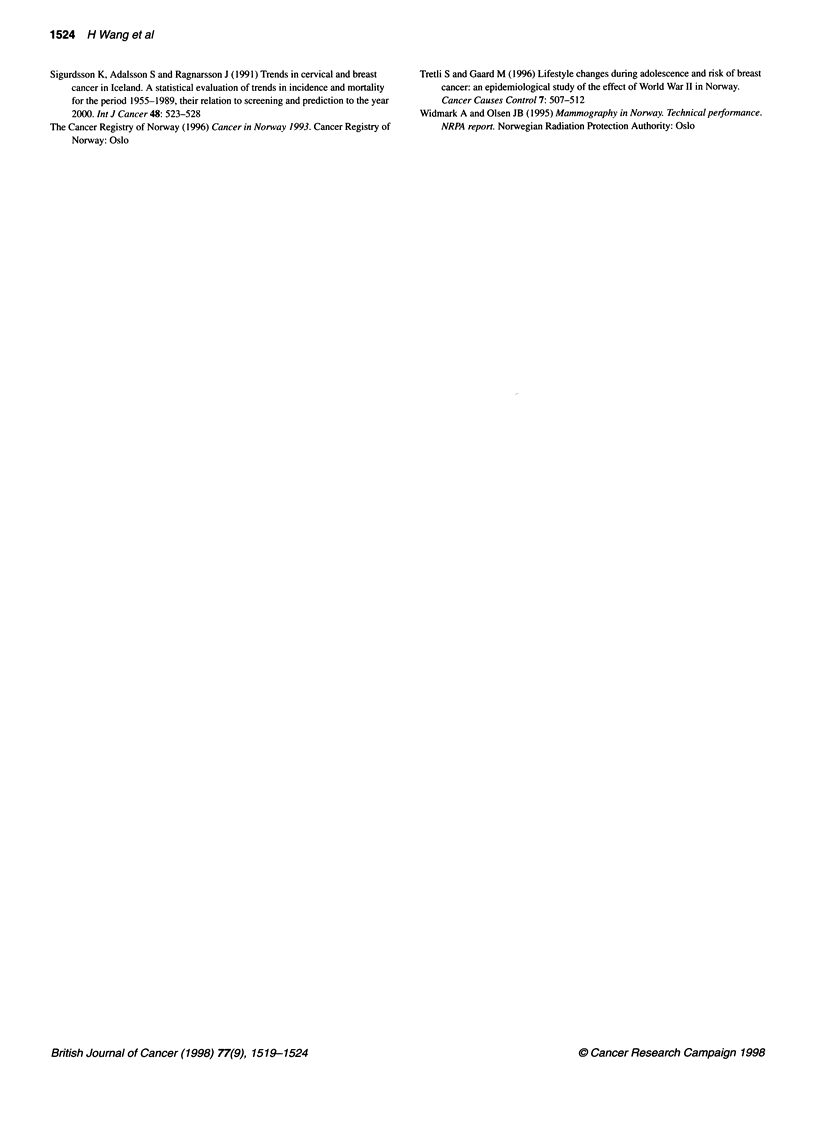# Breast cancer in Norway 1970-1993: a population-based study on incidence, mortality and survival.

**DOI:** 10.1038/bjc.1998.250

**Published:** 1998-05

**Authors:** H. Wang, S. O. Thoresen, S. Tretli

**Affiliations:** The Cancer Registry of Norway, Institute for Epidemiological Cancer Research, Montebello, Oslo.

## Abstract

The incidence, mortality and survival of breast cancer patients from 1970 to 1993 were studied using data from the Cancer Registry of Norway. The age-adjusted incidence rate increased from 62.0 to 76.9 per 100,000 person-years during the period, and more than 2000 cases are now registered annually. The increase tends to be highest in the age group below 40 years. The increase is mainly found in cases with localized tumours at the time of diagnosis. The mortality rate has been almost unchanged in the period; the age adjusted mortality rate is 27.0 per 100,000 person-years at the end of the study period. The 5-year overall survival has increased among cases with axillary lymph node metastases at the time of diagnosis; the other stages show only little improvement.


British Journal of Cancer (1998) 77(9), 1519–1524
© 1998 Cancer Research Campaign

### Breast cancer in Norway 1970–1993: a population-based study on incidence, mortality and survival

**H Wang, SØ Thoresen and S Tretli**

The Cancer Registry of Norway, Institute for Epidemiological Cancer Research, Montebello, N-0310 Oslo, Norway

**Summary** The incidence, mortality and survival of breast cancer patients from 1970 to 1993 were studied using data from the Cancer Registry of Norway. The age-adjusted incidence rate increased from 62.0 to 76.9 per 100 000 person–years during the period, and more than 2000 cases are now registered annually. The increase tends to be highest in the age group below 40 years. The increase is mainly found in cases with localized tumours at the time of diagnosis. The mortality rate has been almost unchanged in the period; the age adjusted mortality rate is 27.0 per 100 000 person–years at the end of the study period. The 5-year overall survival has increased among cases with axillary lymph node metastases at the time of diagnosis; the other stages show only little improvement.

**Keywords:** breast cancer; incidence; stage distribution; mortality; trends

In 1985, the worldwide incidence of breast cancer was estimated to be 720 000 new cases per year, 422 000 in developed countries and 298 000 in developing countries (Parkin et al, 1993). Breast cancer was third in frequency when both sexes were considered together and by far the most important cancer in women (19% of the diagnosis). The incidence rates are increasing in all countries with available statistics, and its impact is magnified because women are at risk from their late thirties. The incidence rates of breast cancer are in general much higher in developed countries than in developing countries; the highest incidence rates are found in North America. In Norway, breast cancer contributed 22.4% of female cancer diagnosis in 1993 (The Cancer Registry of Norway, 1996). Although several risk factors are known, the understanding of breast cancer aetiology is limited. Most of the known risk factors are associated with less than a twofold increase in risk, and breast cancer development is probably a process in which several factors act together.

Mortality rates have generally been increasing worldwide, but the increase is now reported to have declined in several western countries (Hermon and Beral, 1996). This may be explained by earlier detection, better treatment regimes or less aggressive biological behaviour of the tumours. Studies have shown significant mortality reduction by routine mammographic examination of women aged 50–69 years (Nystrøm et al, 1993). Thus, screening programmes have been organized in several European countries. The aim of this study is to describe and analyse the trends of breast cancer incidence in Norway in different age groups and stages together with mortality trends and 5-year relative survival. A large screening project was recently (1996) started in Norway among women aged 50–69 years. It is therefore of interest to analyse breast cancer data in Norway before organized screening was started.

*Received 23 July 1997*
*Revised 30 October 1997*
*Accepted 30 October 1997*

*Correspondence to:* SØ Thoresen

### MATERIALS AND METHODS

The reporting of cancer to the Cancer Registry (CR) of Norway has been compulsory since the registry was established in 1952. The reporting system is based on pathology and cytology reports, clinical records and death certificates. This multiple reporting practice provides an accurate and complete set of data for each patient. Site, histological type, stage of disease at the time of diagnosis, residence and the 11-digit individual identification number allocated to every resident of Norway are reported. The present study is based on 39 006 cases of breast cancer registered from 1970 to 1993.

Incidence and mortality rates are given as rates per 100 000 person–years. The direct method of age standardization with respect to the European standard population was used. The cases were organized into successive 10-year age groups (20–29, 30–39, ... 80 +) and 5-year diagnostic periods where possible. Trends in incidence and mortality rates for 1970–1993 were analysed by fitting a log-linear function. This model was chosen because the curves describe monotonically increasing functions. To have enough data in all groups, the youngest mortality age group was defined as 0–49 years.

Clinical staging was done at the time of diagnosis. According to Cancer Registry definitions, the cases were organized into the clinical stages 1, 2, 3, 4 and 9. Stage 1, tumours of all sizes confined to the breast (except cases belonging to stage 3); stage 2, tumour in the breast with metastases to the axillary lymph nodes; stage 3, tumour in the breast with direct extension to the skin or chest wall (with or without axillary lymph node metastases); stage 4, tumour in the breast with distant metastases; stage 9, unknown. The unknown cases (stage 9) are not included in the statistical analyses, because this small group is a mixture of the other stages.

A multivariate analysis of the 5-year survival was conducted using a model described by Hakulinen and Tenkanen (1987). To have enough data in all groups in this analysis, the youngest age group was defined as 0–39 years. The model was fitted using the GLIM statistical package. The relative risk (RR) of death within 5 years and its 95% confidence interval were computed for each prognostic variable.

Figure 1 Age-adjusted incidence (-◆-) and mortality (-□-) rates in Norway 1970–1993

Figure 2 Stage-specific incidence rates in Norway 1970–1993. (-◆-), Stage 1; (-■-), stage 2; (-▲-), stage 3; (-●-), stage 4; (- ✳ -), stage 9

Table 1 Incidence of breast cancer per 100 000 person-years in Norway 1970–1993 by age and period of diagnosis

| Period | 1970–1974 | 1975–1979 | 1980–1984 | 1985–1989 | 1990–1993 | Estimated percentage annual increase | 95% CI |
|---|---|---|---|---|---|---|---|
| Age group (years) | | | | | | | |
| 0–19 | 0.0 | 0.0 | 0.0 | 0.1 | 0.0 | – | – |
| 20–29 | 2.5 | 2.5 | 3.2 | 2.6 | 3.6 | 1.8 | (0.0, 3.6) |
| 30–39 | 26.4 | 29.7 | 32.4 | 31.0 | 35.9 | 1.4 | (0.7, 2.2) |
| 40–49 | 100.7 | 99.1 | 101.7 | 107.7 | 119.4 | 0.9 | (0.4, 1.3) |
| 50–59 | 121.7 | 139.1 | 130.7 | 136.9 | 148.7 | 0.8 | (0.3, 1.2) |
| 60–69 | 158.8 | 172.8 | 180.8 | 190.3 | 191.5 | 1.0 | (0.7, 1.3) |
| 70–79 | 188.0 | 221.7 | 238.9 | 244.7 | 248.6 | 1.3 | (1.0, 1.7) |
| 80+ | 226.6 | 244.9 | 272.1 | 289.1 | 281.2 | 1.2 | (0.7, 1.7) |
| Crude rate | 66.0 | 74.0 | 79.0 | 84.5 | 89.2 | | |
| Age adjusted | 62.0 | 68.0 | 69.8 | 72.9 | 76.9 | 1.0 | (0.8, 1.2) |
| Number of cases/year | 1309 | 1509 | 1643 | 1792 | 1932 | | |

© Cancer Research Campaign 1998

**Table 2** Incidence numbers by period of diagnosis and stage

| Period | 1970–1974 | 1975–1979 | 1980–1984 | 1985–1989 | 1990–1993 |
|---|---|---|---|---|---|
| | *n* (%) | *n* (%) | *n* (%) | *n* (%) | *n* (%) |
| Stage | | | | | |
| 1 | 3262 (49.8) | 4060 (53.8) | 4640 (56.5) | 5152 (57.5) | 4525 (58.5) |
| 2 | 2080 (31.8) | 2205 (29.2) | 2458 (29.9) | 2634 (29.4) | 2328 (30.1) |
| 3 | 485 (7.4) | 579 (7.7) | 464 (5.7) | 429 (4.8) | 254 (3.3) |
| 4 | 558 (8.5) | 580 (7.7) | 526 (6.4) | 630 (7.0) | 507 (6.6) |
| 9 | 164 (2.5) | 122 (1.6) | 131 (1.6) | 116 (1.3) | 119 (1.5) |
| Total | 6549 (100.0) | 7546 (100.0) | 8219 (100.0) | 8961 (100.0) | 7733 (100.00) |

**Table 3** Proportion of tumours at different stages and age groups in the periods 1970–1974 and 1990–1993

| Period | 1970–1974 | | | | | 1990–1993 | | | | |
|---|---|---|---|---|---|---|---|---|---|---|
| Stage | 1<br>% (*n*) | 2<br>% (*n*) | 3<br>% (*n*) | 4<br>% (*n*) | 9<br>% (*n*) | 1<br>% (*n*) | 2<br>% (*n*) | 3<br>% (*n*) | 4<br>% (*n*) | 9<br>% (*n*) |
| Age groups (years) | | | | | | | | | | |
| 0–39 | 52.0 (157) | 39.4 (119) | 4.0 (12) | 4.6 (14) | 3.0 (5) | 50.4 (245) | 41.2 (200) | 1.4 (7) | 7.0 (34) | 1.7 (2) |
| 40–49 | 53.5 (585) | 36.5 (399) | 4.8 (53) | 5.2 (57) | 10.9 (18) | 55.1 (761) | 37.9 (523) | 1.6 (22) | 5.4 (74) | 7.8 (9) |
| 50–59 | 47.6 (703) | 36.4 (538) | 6.4 (94) | 9.6 (142) | 12.1 (20) | 56.1 (651) | 36.0 (418) | 1.8 (21) | 6.1 (70) | 6.1 (7) |
| 60–69 | 49.2 (797) | 34.4 (558) | 8.0 (129) | 8.4 (136) | 20.6 (34) | 56.7 (877) | 33.0 (510) | 2.9 (45) | 7.4 (115) | 9.6 (11) |
| 70+ | 53.9 (1020) | 24.6 (466) | 10.4 (197) | 11.1 (209) | 53.4 (88) | 64.9 (1982) | 22.6 (690) | 5.3 (161) | 7.2 (221) | 74.8 (86) |

The urban/rural status is defined by 'Statistics Norway' on the basis of administrative areas. The difference between urban and rural incidence and mortality rates was analysed by dividing the study period into two periods: 1970–1981 and 1982–1993.

### RESULTS

### Incidence

The age-adjusted incidence and mortality curves are shown in Figure 1. The average annual number of breast cancer cases increased from 1309 in the period 1970–1974 to 1932 in the period 1990–1993, a 47% increase (Table 1). There is an increasing incidence rate in all age groups during the whole study period. The annual age-adjusted increase in the incidence rate is estimated at 1.0% (Table 1). The increase tends to be higher in the youngest (< 40 years) and oldest (> 70 years) age groups. In the latest decade (1984–1993), the annual increases were estimated to be 3.3%, 2.7% and 2.2% for the age groups 0–29, 30–39 and 40–49 years respectively (data not shown).

Stage-specific incidence rates by period are illustrated in Figure 2, and absolute numbers are presented in Table 2. Table 3 presents the proportion of tumours at different stages and ages in the periods 1970–1974 and 1990–1993. The increasing incidence is mainly found in stage 1; the total number of cases in stage 1 increased from 3262 in 1970–1974 to 5152 in 1985–1989. In women older than 80 years, 65.4% were diagnosed as stage 1 in 1985–1989. This is an increase of more than 12% since 1970–1974. The number of cases in this age group increased from 342 to 930 in the two periods.

The urban/rural analysis reveals that the age-adjusted relative incidence rate tends to be almost unchanged, with an urban dominance decreasing only from 1.21 to 1.16. Among women below 40 years, there was no difference in urban/rural incidence rates in 1982–1993, indicating that the increase in incidence has been higher in rural than in urban areas.

### Mortality

The age-adjusted mortality rate is practically unchanged during the study period (Figure 1), with an estimated yearly increase of 0.5%. There is a small reduction in the age group 50–59 years (– 0.6%), whereas the mortality rates increase most among women above 80 years (1.6%) (Table 4). The urban/rural analysis (data not shown) reveals that the relative mortality rate tends to be almost unchanged, with an urban dominance decreasing from 1.17 to 1.10. Among women under 60 years, no difference was found in urban/rural incidence rates in 1982–1993.

### Survival

The 5-year relative survival rates by age and stage in the two periods 1970–1974 and 1985–1989 are shown in Table 5. The survival rates in stage 2 have increased for all ages; the other stages show only little improvement. The highest 5-year survival rate, 90.8%, is found in the age group 40–49 years diagnosed as stage 1 in the latest period.

The multivariate analysis with three variables (age, stage and period) gave an acceptable fit. The estimates of relative risk (RR) of death within 5 years and their 95% confidence interval are given in Table 6. As expected, stage at time of diagnosis was found to be the most important prognostic factor. All age groups are found to have slightly higher RR than the reference group 40–49 years. The RR is reduced by 20% in the latest period, indicating a better overall survival.

**Table 4** Mortality of breast cancer per 100 000 person-years in Norway 1970–1993 by age and period of diagnosis

| Period | 1970–1974 | 1975–1979 | 1980–1984 | 1985–1989 | 1990–1993 | Estimated percentage annual increase | 95% CI |
|---|---|---|---|---|---|---|---|
| Age group (years) | | | | | | | |
| 0–49 | 5.1 | 4.4 | 4.2 | 5.8 | 7.0 | 1.8 | (0.2, 3.3) |
| 50–59 | 52.9 | 54.9 | 50.6 | 52.1 | 45.2 | –0.6 | (–1.0, –0.2) |
| 60–69 | 67.5 | 77.1 | 73.3 | 74.8 | 76.3 | 0.4 | (–0.1, 0.9) |
| 70–79 | 89.4 | 100.3 | 106.6 | 112.1 | 107.7 | 1.1 | (0.4, 1.8) |
| 80+ | 132.9 | 145.1 | 153.9 | 175.6 | 177.3 | 1.6 | (1.1, 2.1) |
| Crude rate | 27.3 | 30.3 | 31.0 | 34.3 | 34.3 | | |
| Age adjusted | 24.4 | 25.9 | 25.1 | 27.0 | 26.4 | 0.5 | (0.2, 0.7) |
| Number of cases/year | 541 | 618 | 645 | 727 | 746 | | |

**Table 5** Five-year relative survival rates (%) in patients with breast cancer in Norway, by stage, age and period of diagnosis

| Period | 1970–1974 | | | | 1985–1989 | | | |
|---|---|---|---|---|---|---|---|---|
| Stage | 1 | 2 | 3 | 4 | 1 | 2 | 3 | 4 |
| Age groups (years) | | | | | | | | |
| 0–39 | 85.3 | 54.2 | 33.3 | 28.6 | 87.3 | 59.8 | 40.0 | 21.7 |
| 40–49 | 90.4 | 63.8 | 47.2 | 21.1 | 90.8 | 68.9 | 46.7 | 13.6 |
| 50–59 | 82.3 | 55.9 | 45.7 | 7.7 | 85.7 | 62.0 | 52.9 | 7.4 |
| 60–69 | 80.4 | 51.0 | 46.5 | 9.7 | 85.3 | 66.1 | 64.9 | 11.8 |
| 70+ | 53.0 | 37.3 | 29.6 | 10.1 | 58.6 | 50.4 | 34.4 | 11.4 |

**Table 6** Relative risk (RR) of death within 5 years and 95% confidence intervals in breast cancer patients according to stage, period of diagnosis and age

| Variable | RR | 95% CI |
|---|---|---|
| Age group (years) | | |
| 0–39 | 1.4 | (1.1, 1.7) |
| 40–49 | 1.0 | Reference group |
| 50–59 | 1.3 | (1.1, 1.6) |
| 60–69 | 1.1 | (1.0, 1.4) |
| 70+ | 1.2 | (1.0, 1.5) |
| Stage | | |
| 1 | 1.0 | Reference group |
| 2 | 3.7 | (3.2, 4.3) |
| 3 | 5.2 | (4.2, 6.4) |
| 4 | 16.1 | (13.5, 19.1) |
| Period | | |
| 1970–1974 | 1.0 | Reference group |
| 1985–1989 | 0.8 | (0.7, 0.9) |

### DISCUSSION

In the present study, all cases of breast cancer reported to the Cancer Registry of Norway during 1970–1993 were subjected to an analysis of incidence, mortality and survival. The completeness of the Cancer Registry data, both on diagnosis and on cause of death, make the data reliable. The main finding in this study is the increasing incidence rate during the whole study period, estimated to be 1.0% yearly. Estimates of annual increase are higher for the last decade than for the whole study period. Increasing incidence rates are reported in Denmark and Iceland before organized mammography screening (Andreasen et al, 1994; Sigurdsson et al, 1991). Some studies from the USA (Newcomb and Lantz, 1993; Garfinkel et al, 1994) show declining incidence rates since the late 1980s after several years of increasing incidence. This is probably due to more years of extended use of mammography in asymptomatic women. Norway, however, has had no organized screening programme for breast cancer during the study period. Still, the yearly number of mammograms taken unorganized in Norway has increased from 10 000 in 1983 to 220 000 in 1993 (Widmark and Olsen, 1995). Most of these mammograms were taken among younger women (therefore at lower risk) living in urban areas.

We found that incidence rates are increasing in all age groups, but the increase is most pronounced among women younger than 40 years. As the increase in these age groups is highest in rural areas, this increase is probably not caused by extended use of mammography. A similar trend in incidence among women younger than 40 was not found in Sweden (Persson et al, 1998), but our results confirm a previous report on incidence increase among Norwegian women under 50 years in the period 1983–1993 (Matheson and Tretli, 1996). A transient incidence increase reported among Swedish women aged 50–69 years is most probably the result of mammographic screening (Persson et al, 1998). It will be interesting to see if the ongoing Norwegian screening project will cause a similar transient incidence increase among women aged 50–69 years. A Danish study (Andreasen et al, 1994), before mammographic screening, reports that the largest increase in incidence was found among women younger than 60 years. In some countries of low incidence, such as Japan, the rise in incidence has so far been observed predominantly in women under the age of 50 years and appears to be a birth cohort effect (Miller and Bulbrook, 1986). Lower breast cancer incidence rates than

 © Cancer Research Campaign 1998

expected are reported among Norwegian women who experienced their adolescence during World War II (Tretli and Gaard, 1996). This birth cohort effect implies that one or more lifestyle factors that changed among adolescent women during the war influenced their risk of breast cancer. There is an increasing awareness of the importance of the period between menarche and first birth (Colditz and Frazier, 1995). Factors such as adolescence nutrition (Tretli and Gaard, 1996) and regular physical exercise (Bernstein et al, 1994) are thought to have an impact on later risk of developing breast cancer, but more studies are required on the possible impact of teenage lifestyle on breast cancer risk.

Attention has focused an environmental and dietary oestrogens as possible contributors to the increased incidence of breast cancer but the epidemiological findings are inconclusive (Ahlborg et al, 1995; Safe, 1995). However, even though the total amount of environmental and dietary oestrogen is low, it might have an influence over time, especially in adolescent women. It is well known that a woman's reproductive history affects her risk of breast cancer (Kvåle et al, 1987; Kvåle and Heuch, 1987). The delayed child-bearing pattern that is seen in most western countries is another factor that may contribute to a woman's total risk of developing breast cancer. These factors may be contributors to the observed increase in incidence rates among younger women. It is interesting to notice that the difference between urban and rural areas is decreasing and, among younger women, no difference in incidence rate is found in the latest period. This may be explained by the fact that rural lifestyle, including nutrition, has changed towards that in urban areas.

Our data reveal an excess increase in incidence rates among older women (above 70 years). This may be explained by an increasing proportion of very elderly women together with increased breast cancer awareness. The increase in incidence in stage 1 diagnosis is especially high in this age group, but this is probably an artifact caused by less extensive examination of axillary lymph nodes among older patients.

The role of stage 1 diagnosis is increasing (Figure 2 and Table 3). There may be several reasons for this increase in stage 1 diagnosis, for instance early detection or change in tumour biology. Mammography of asymptomatic women is a method that causes stage migration towards stage 1, but in Norway most women detect their breast cancer themselves. The increased breast cancer awareness in general and instruction in self-palpation have caused women to visit their doctor earlier, thus getting their diagnosis at a stage with better prognosis. It is likely, however, that as a result of the ongoing large screening project, the part of stage 1 diagnosis will continue to increase in the age group 50–69 years. One may speculate that change in some environmental factors initiates tumour development more easily than before, but that the tumours are less aggressive and remain longer in stage 1. A tendency for oral contraceptive users to have more localized tumours than never users has been reported (Collaborative group on Hormonal Factors in Breast Cancer, 1996) and perhaps other factors such as xeno-oestrogens have a similar effect.

The possibility has been raised that lesions that would remain benign are now being diagnosed and treated as cancer. If so, we would have expected a more pronounced improvement in 5-year survival among the stage 1 cases. It is, therefore, more likely that the cases diagnosed would develop into stages with poorer prognosis if not treated properly.

We find that the mortality rate is practically unchanged in the study period. It is interesting to note that the Scandinavian countries (which all have complete cancer registers) report different mortality trends. Denmark reports an unchanged mortality rate, as in this study (Ewertz and Carstensen, 1988). In Finland, the mortality was increasing until 1985, whereas Sweden has had a small decline since 1975 (Hermon and Beral, 1996). It is difficult to explain which differences in lifestyle or health system might cause these different mortality trends, although nutrition and use of mammography could be among relevant factors.

As expected, stage at time of diagnosis is found to be the most important factor influencing the relative risk of death within 5 years. The multivariable analysis shows that this risk has significantly reduced during the study period by 20% (Table 6), indicating better survival especially in stage 2, probably as a result of better treatment. This is also reflected in the 5-year survival rates in Table 5. In the 1970s, surgery and radiation were the main treatment regimes, whereas chemotherapy has played a larger role in recent years. The use of such treatment and hormones have probably had a positive impact on survival.

### REFERENCES

Ahlborg U, Lipworth L, Titus-Ernstoff L, Hsieh C and Baron J (1995) Organochlorine compounds in relation to breast cancer, endometrial cancer and endometriosis: an assessment of the biological and epidemiological evidence. *Crit Rev Toxicol* **25**: 463–531

Andreasen AH, Andersen KW, Madses M, Mouridsen H, Olesen KP and Lynge E (1994) Regional trends in breast cancer incidence and mortality in Denmark prior to mammographic screening. *Br J Cancer* **70**: 133–137

Bernstein L, Henderson BE, Hanisch R, Sullivan-Halley J and Ross K (1994) Physical exercise and reduced risk of breast cancer in young women. *J Natl Cancer Inst* **86**: 1403–1408

Colditz GA and Frazier AL (1995) Models of breast cancer show that risk is set by events of early life: prevention effects must shift focus. *Cancer Epidemiol Biomarkers Prev* **4**: 567–571

Collaborative group on Hormonal Factors in Breast Cancer (1996) Breast cancer and hormonal contraceptives: collaborative reanalysis of individual data on 53 297 women with breast cancer and 100 239 women without breast cancer from 54 epidemiological studies. *Lancet* **347**: 1713–1727

Ewertz M and Carstensen B (1988) Trends in breast cancer incidence and mortality in Denmark 1943–1982. *Int J Cancer* **41**: 46–51

Garfinkel L, Boring C and Heath CW (1994) Changing trends – an overview of breast cancer incidence and mortality. *Cancer* **74**: 222–226

Hakulinen T and Tenkanen L (1987) Regression analysis of relative survival rates. *Appl Stat* **3**: 309–317

Hermon C and Beral V (1996) Breast cancer mortality rates are levelling off or beginning to decline in many western countries: analysis of time trends, age, cohort and age-period models of breast cancer mortality in 20 countries. *Br J Cancer* **73**: 955–960

Kvåle G and Heuch I (1987) A prospective study of reproductive factors and breast cancer II. Age at first and last birth. *Am J Epidemiol* **126**: 842–850

Kvåle G, Heuch I and Eide GE (1987) A prospective study of reproductive factors and breast cancer I. Parity. *Am J Epidemiol* **126**: 831–841

Matheson I and Tretli S (1996) Changes in breast cancer incidence among Norwegian women under 50. *Lancet* **348**: 900–901

Miller AB and Bulbrook RD (1986) UICC multidisciplinary project on breast cancer: the epidemiology, aetiology and prevention of breast cancer. *Int J Cancer* **37**: 173–177

Newcomb PA and Lantz P (1993) Recent trends in breast cancer incidence, mortality and mammography. *Breast Cancer Res Treat* **28**: 97–106

Nystrøm L, Rutqvist LE, Wall S, Lindgren A, Lindqvist M, Ryden S, Andersson I, Burstam N, Fagerberg G, Frisell J, Tabar L and Larsson L-G (1993) Breast cancer screening with mammography: overview of Swedish randomized studies. *Lancet* **341**: 973–978

Parkin DM, Pisani P and Fearly J (1993) Estimates of the world-wide incidence of eighteen major cancers in 1985. *Int J Cancer* **54**: 594–606

Persson I, Bergström R, Lotti B and Adami H-O (1998) Recent trends in breast cancer incidence in Sweden. *Br J Cancer* **77**: 167–169

Safe S (1995) Environmental and dietary estrogens and human health: is there a problem? *Environ Health Perspect* **103**: 346–351

© Cancer Research Campaign 1998

Sigurdsson K, Adalsson S and Ragnarsson J (1991) Trends in cervical and breast cancer in Iceland. A statistical evaluation of trends in incidence and mortality for the period 1955–1989, their relation to screening and prediction to the year 2000. *Int J Cancer* **48**: 523–528

The Cancer Registry of Norway (1996) *Cancer in Norway 1993*. Cancer Registry of Norway: Oslo

Tretli S and Gaard M (1996) Lifestyle changes during adolescence and risk of breast cancer: an epidemiological study of the effect of World War II in Norway. *Cancer Causes Control* **7**: 507–512

Widmark A and Olsen JB (1995) *Mammography in Norway. Technical performance. NRPA report.* Norwegian Radiation Protection Authority: Oslo

*© Cancer Research Campaign 1998*